# Review of the genus *Apotrechus* in China (Orthoptera, Gryllacrididae, Gryllacridinae)

**DOI:** 10.3897/zookeys.482.8713

**Published:** 2015-02-16

**Authors:** Miao-Miao Li, Xian-Wei Liu, Kai Li

**Affiliations:** 1 School of Life Science, East China Normal University, Shanghai 200241, China; 2 Shanghai Entomology Museum, Chinese Academy of Sciences, Shanghai 200032, China

**Keywords:** Gryllacrididae, Gryllacridinae, *Apotrechus*, new species, China

## Abstract

In the present paper, the genus *Apotrechus* Brunner-Wattenwyl, 1888 is revised. Two new species from China are described and illustrated: *Apotrechus quadratus* **sp. n.** and *Apotrechus truncatolobus* **sp. n..** A new key and the distributional data are given.

## Introduction

The genus *Apotrechus* was proposed by Brunner-Wattenwyl (1888), with the type species *Apotrechus unicolor* Brunner-Wattenwyl, 1888. This genus resembles the genus *Eremus* Brunner-Wattenwyl, 1888, but differs from the latter in: smooth frons, spineless hind tibia and absence of male styli. Liu and Yin (2002) first studied *Apotrechus* in China, described one new species *Apotrechus nigrigeniculatus*. Liu and Bi (2008) gave a key of *Apotrechus* from China containing three species, and two new species *Apotrechus digitatus* and *Apotrechus fallax* were illustrated. Besides, Liu et al. (2010) also reported one new species *Apotrechus transversus* from Zhejiang. Subsquently, Guo and Shi (2012) reviewed this genus of China and also provided a key containing six species in China which included one new species *Apotrechus bilobus*, and one new combination *Apotrechus parvospinus*.

Bian et al. (2014) provided a key to the species with one new species *Apotrechus trilobus* and the morphological photographs for five Chinese known species in this paper.

So far, the genus *Apotrechus* includes nine species in the world, among them, *Apotrechus unicolor* Brunner-Wattenwyl, 1888, *Apotrechus swinhoei* (Griffini, 1909), and *Apotrechus illawarra* Rentz, 1990 are recorded in Australia; *Apotrechus insolitus* (Walker, 1869) is distributed in Vietnam and others are recorded in China. In this paper, tow new species of *Apotrechus* are identified and described, namely *Apotrechus quadratus* sp. n. and *Apotrechus truncatolobus* sp. n., which are distributed in Guangxi.

## Material and methods

All specimens of the genus were collected by light-trapping and net-catching from China. Adult specimens were preserved in 70% ethanol in the field, then removed and dried in the lab. The specimens were observed with the help of a Leica MZ 12.5 dissecting microscope and illustrated with the aid of a drawing tube attached to the microscope. Line drawings were made with Adobe Illustrator CS 6 graphic software. The length of the body was measured mesaby the distance between apex of fastigium verticis and posterior margin of tenth abdominal tergite, ovipositor by distance between base of subgenital plate and apex of ovipositor; pronotum, tegmina and hind femora by distance between summit of base and apex. All lengths are presented in millimeters. The venation nomenclature used in this paper is based on the interpretation of Karny (1937). All type specimens recorded here are deposited in the Shanghai Entomology Museum, the Chinese Academy of Sciences.

## Taxonomy

### Genus Apotrechus

Taxon classificationAnimaliaOrthopteraGryllacrididae

Brunner-Wattenwyl, 1888

urn:lsid:orthoptera.speciesfile.org:TaxonName:21786

Apotrechus : Brunner-Wattenwyl 1888: 383; Tepper 1892: 167; Kirby 1906: 152; Ramme 1933: 416; Karny 1937: 82; Rentz and John 1990: 1083; Liu and Bi 2008: 11, figs 1–5; Liu et al. 2010: 64; Guo and Shi 2012: 52.

#### Type species.

*Apotrechus unicolor* Brunner-Wattenwyl, 1888.

#### Generic diagnosis.

Body small, wings absent. Fastigium of vertex rather wide than scape, without lateral carinae; frons smooth, ocelli inconspicuous. Fore and mid tibiae with 4–5 pairs of spurs on ventral surface, mid tibia without inner upper apical spur on dorsal surface. Hind tibia armless or with rather small spine on ventral surface. Subgenital plate of male without styli. Ovipositor rather short, upcurved.

#### Key to the Chinese species of the genus *Apotrechus*

**Table:** 

1	Fore and mid femora with black apical part	**2**
–	Fore and mid femora without black apical part	**5**
2	External margin of hind femur without spine; lobes of male subgenital plate with acute apex	***Apotrechus trilobus* Bian & Shi, 2014**
–	External margin of hind femur with spines	**3**
3	Body smaller, about 14–18 mm long	**4**
–	Body larger, about 23 mm long; hind margin of female subgenital plate slightly concave	***Apotrechus quadratus* sp. n.**
4	Male 9^th^ abdomenal tergite deeply excised; hind margin of female subgenital plate truncated	***Apotrechus nigrigeniculatus* Liu & Yin, 2002**
–	Male 9^th^ abdomenal tergite shallowly excised; hind margin of female subgenital plate rounded	***Apotrechus fallax* Liu & Bi, 2008**
5	Frons without blackish longitudinal stripe; lobes of male 9^th^ abdomenal tergite with roundly truncate apex	***Apotrechus truncatolobus* sp. n.**
–	Frons with 2–3 blackish longitudinal stripes; lobes of male 9^th^ abdomenal tergite with acute apex	**6**
6	Frons smooth	**7**
–	Frons sunken; dorsal side of hind tibia armed with 3 external and 2 internal spines	***Apotrechus parvospinus* (Liu & Yin, 2002)**
7	Frons with 2 blackish longitudinal stripes; male subgenital plate with incurved lobes; female subgenital plate a bit broader than long	**8**
–	Frons with 3 blackish longitudinal stripes; male subgenital plate with straight lobes; female subgenital plate transverse, ovipositor with lateral lobes at base	***Apotrechus transversus* Liu et al., 2010**
8	Lobes of male subgenital plate with finger-shaped apex; ovipositor without lateral lobes at base	***Apotrechus digitatus* Liu & Bi, 2008**
–	Lobes of male subgenital plate with broadly rounded apex; ovipositor with lateral lobes at base	***Apotrechus bilobus* Guo & Shi, 2012**

### 1. Apotrechus trilobus

Taxon classificationAnimaliaOrthopteraGryllacrididae

Bian & Shi, 2014

http://zoobank.org/4DA1028E-49F8-4F99-A0ED-F632A4BFC4C3

Figs 1–6

Apotrechus trilobus : Bian et al. 2014: 384–386.

#### Description.

Male. Body medium sized. Wings absent. Fastigium of vertex rounded, about 2 times as wide as scape; eyes reniform, prominent; ocelli inconspicuous. Pronotum almost hexagon, paranota lower. Fore coxa with a spine, fore tibia on ventral surface with 5 pairs of spurs (included 1 pair of apical spurs); mid tibia without inner upper apical spur but with 4 pairs of spurs (included 1 pair of apical spurs) on ventral surface. Ventral surface of hind femur with 10–11 internal spines, but without external spine, hind tibia unarmed or with 1–2 minute spines on dorsal surface, bearing 3 pairs of apical spurs. 9^th^ abdominal tergite divided into two lobes, which bearing spine-like apex pointing downwards, epiproct medially furrowed (Fig. 5). Cerci shorter, conical; subgenital plate broad, hind margin split into two lobes, apex of lobes spine-like, curved inside (Fig. 6).

**Figures 1–6.**
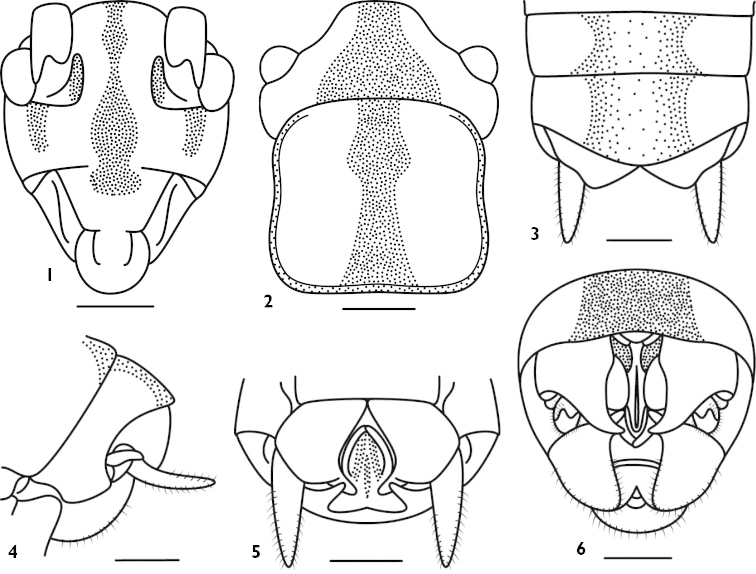
*Apotrechus trilobus* Bian & Shi, 2014. **1** head in frontal view **2** head and pronotum in dorsal view **3** end of male abdomen in dorsal view **4** end of male abdomen in lateral view **5** end of male abdomen in ventral view **6** end of male abdomen in caudal view. Scale: 1 mm.

Female. Unknown.

#### Coloration.

Body infuscate. Fastigium of vertex with darkish black longitudinal band; frons with 3 blackish longitudinal stripes, middle stripe broad, not connected with the longitudinal band of fastigium of vertex (Figs 1–2); inner margin of antenna foveolae and first segment with blackish spots. Pronotum with a darkish black longitudinal band in the middle and all margins black. Apex of fore and middle femora black, hind femur with a blackish longitudinal stripe on external surface, all tibiae darkish black on the base and apex.

#### Measurements.

(length in mm)

**Apotrechus trilobus:** Measurements (length in mm)

	Body	Pronotum	Hind femur	Ovipositor
♂	16.0	3.8	10.0	-

#### Material.

1♂, Yunnan, Pingbian, Yuping, 2000m, 20.V.2009, Xian-Wei Liu et al. leg.

#### Distribution.

China: Yunnan.

### 2. Apotrechus quadratus

Taxon classificationAnimaliaOrthopteraGryllacrididae

Li & Liu sp. n.

http://zoobank.org/F5255058-CCB5-4734-9157-89E36EB98A9E

Figs 7–9

#### Description.

Female. Body large. Wings apterous. Fastigium of vertex roundly projected, about 2 times as wide as scape; eyes reniform, produced; ocelli faintly. Pronotum almost hexagon, lateral lobes longer than high. Fore coxa with a spine, fore tibia on ventral surface with 5 pairs of spurs (included 1 pair of apical spurs) but without inner upper apical spur; mid tibia on ventral surface with 4 pairs of spurs (included 1 pair of apical spurs). Hind femur on ventral surface armed 8 internal spines and 1–3 external spines; hind tibia on dorsal surface bearing 6 pairs of rather small spines and 2 pairs of apical spurs. Cerci shorter, conical; subgenital plate broad, square, and hind margin slightly concave (Fig. 9). Ovipositor short, curved upwards, apex blunt.

**Figures 7–9.**
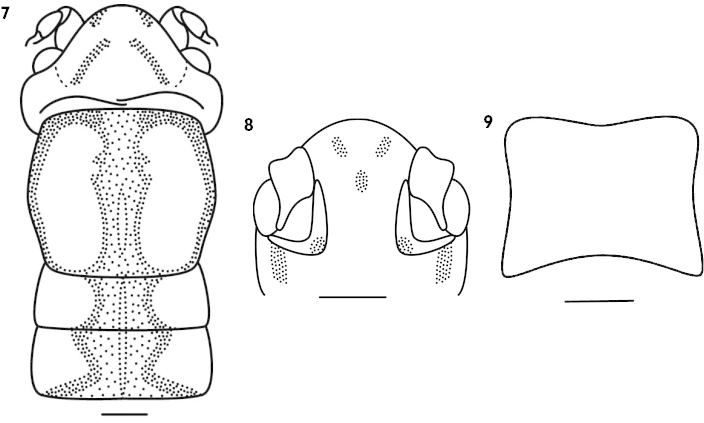
*Apotrechus quadratus* sp. n. **7** head and pronotum in dorsal view **8** frons in front view **9** subgenital plate of female in ventral view. Scale: 1 mm.

Male. Unknown.

#### Coloration.

Body yellowish brown. Fastigium of vertex with 2 pairs of darkish black longitudinal bands; frons with 3 blackish longitudinal spots; inner margin of basal antenna and first segment with blackish spots. Lateral and fore margin of pronotum black, in the middle with a darkish black vertical stripe. Mesonotum and metanotum also with a black spot at middle parts (Figs 7–8). Hind femur with a blackish longitudinal stripe on external surface, all tibiae on base and apex darkish black.

#### Measurements.

(length in mm)

**Apotrechus quadratus:** Measurements (length in mm)

	Body	Pronotum	Hind femur	Ovipositor
♀	23.0	4.8	10.5	5.5

#### Material.

Holotype ♀, Guangxi, Xing’an, Maoer Mountain, 1700–2100m, 30.VII–6.VIII. 2013, Xian-Wei Liu et al. leg.

#### Distribution.

China: Guangxi.

#### Diagnosis.

This new species is closely related to *Apotrechus nigrigeniculatus* Liu & Yin, 2002, but differs mainly in the the latter in body larger and subgenital plate of female with hind margin slightly concave.

#### Etymology.

The specific epithet referrers to shape of female subgenital plate.

### 3. Apotrechus nigrigeniculatus

Taxon classificationAnimaliaOrthopteraGryllacrididae

Liu & Yin, 2002

urn:lsid:orthoptera.speciesfile.org:TaxonName:21789

Figs 10–11

Apotrechus nigrigeniculatus : Liu and Yin 2002: 418; Guo and Shi 2012: 53; Bian et al. 2014: 383.

#### Measurements.

(length in mm)

**Apotrechus nigrigeniculatus:** Measurements (length in mm)

	Body	Pronotum	Hind femur	Ovipositor
♂	15.0–16.5	3.5	9.0–10.0	-
♀	14.0	3.7	7.5	5.0

#### Material.

2♂♂, Sichuan, Emei Mountain, 1840m, 16.VIII.1985, Gen-Tao Jin leg..

**Figures 10–11.**
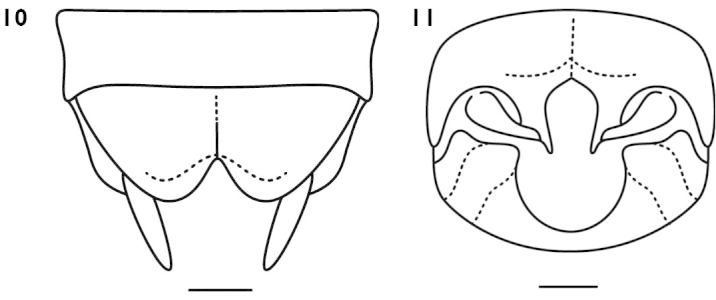
*Apotrechus nigrigeniculatus* Liu & Yin, 2002. **10** end of male abdomen in dorsal view **11** end of male abdomen in caudal view. Scale: 1 mm.

#### Distribution.

China: Sichuan.

### 4. Apotrechus fallax

Taxon classificationAnimaliaOrthopteraGryllacrididae

Liu & Bi, 2008

urn:lsid:orthoptera.speciesfile.org:TaxonName:21787

Figs 12–16

Apotrechus fallax : Liu and Bi 2008: 13, figs 1–5; Guo and Shi 2012: 53; Bian et al. 2014: 382.

#### Measurements.

(length in mm)

**Apotrechus fallax:** Measurements (length in mm)

	Body	Pronotum	Hind femur	Ovipositor
♂	14.0	3.8	8.0	-
♀	18.0	3.8	8.0	5.5

#### Material.

1♀, Guizhou, Leigongshan, 1620–2178m, 2.VIII.2004, Pian Xu leg.; 1♂, Guizhou, Leigong Mountain, 1000–1100m, 2–3.VI.2005, Zheng-Guang Zhang leg.; 2♂♂, Guizhou, Jiangkou, Fanjingshan, 1200–1800m, 6.VIII.2014, Miao-Miao Li & Mei-Ling Sun leg..

**Figures 12–16.**
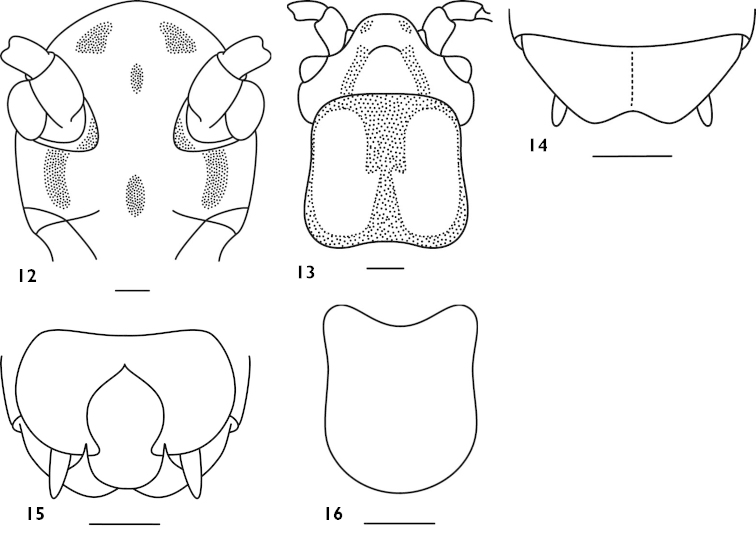
*Apotrechus fallax* Liu & Bi, 2008. **12** head in frontal view **13** head and pronotum in dorsal view **14** end of male abdomen in dorsal view **15** end of male abdomen in ventral view **16** subgenital plate of female in ventral view. Scale: 1 mm.

#### Distribution.

China: Guizhou.

### 5. Apotrechus truncatolobus

Taxon classificationAnimaliaOrthopteraGryllacrididae

Li & Liu sp. n.

http://zoobank.org/76C1D15F-B014-454D-855A-365A94B4E267

Figs 17–21

#### Description.

Male. Body medium sized. Wings absent. Fastigium of vertex rounded, about 2 times as wide as scape; eyes ovoid, prominent, ocelli inconspicuous. Cephalic margin of pronotum slightly projected, posterior margin slightly truncated, lateral lobes lower. Fore coxa with a spine, fore tibia on ventral surface with 5 pairs of spurs (included 1 pair of apical spurs); mid tibia without inner upper apical spur but on ventral surface with 4 pairs of spurs (included 1 pair of apical spurs); hind tibia without spine or on dorsal surface with 1–2 minute spines, with 3 pairs of apical spurs. Hind femur with 10–12 internal spines and 7–8 external spines on ventral surface. Lobes of 9^th^ abdominal tergite with roundly truncated apex (Fig. 20); cerci shorter, conical; subgenital plate broad, hind margin split into two lobes and with notch in the middle (Figs 18–19).

**Figures 17–21.**
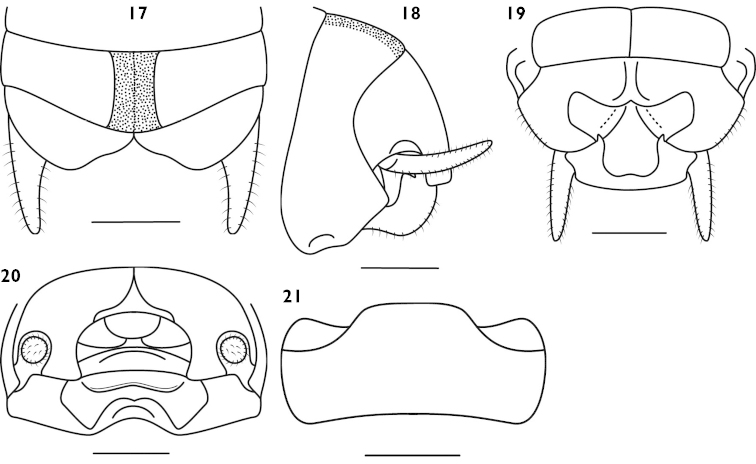
*Apotrechus truncatolobus* sp. n. **17** end of male abdomen in dorsal view **18** end of male abdomen in lateral view **19** end of male abdomen in ventral view **20** end of male abdomen in caudal view **21** subgenital plate of female in ventral view. Scale: 1 mm.

Female. Cerci short, conical; subgenital plate strongly transverse, with straight hind margin and rounded posterio-lateral corner (Fig. 21). Ovipositor shorter than hind tibia, upcurved and with blunt apex.

#### Coloration.

Body yellowish brown, occiput slightly with darkish black. Frons without blackish longitudinal stripes; dorsal margin of abdominal with a darkish black longitudinal band in the middle (Fig. 17). Apex of fore femur, base and apex of tibiae slightly darkish black.

#### Measurements.

(length in mm)

**Apotrechus truncatolobus:** Measurements (length in mm)

	Body	Pronotum	Hind femur	Ovipositor
♂	16.0	3.5	9.0	-
♀	18.0	3.8	9.0	6.0

#### Material.

Holotype ♂, paratype 1♂1♀, Guangxi, Wuming, Daming Mountain, 1200m, 28–31.VII.2012, Wen-Xuan Bi leg.

#### Distribution.

China: Guangxi.

#### Diagnosis.

This new species almost the same as its congeners, but the frons without blackish longitudinal stripe; lobes of male 9^th^ abdominal tergite with roundly truncate apex.

#### Etymology.

The specific epithet referrers roundly truncate lobes of male 9^th^ abdominal tergite.

### 6. Apotrechus parvospinus

Taxon classificationAnimaliaOrthopteraGryllacrididae

(Liu & Yin, 2002)

urn:lsid:orthoptera.speciesfile.org:TaxonName:73813

Figs 22–24

Eremus parvospinus : Liu and Yin 2002: 417.
Apotrechus parvospinus : Guo and Shi 2012: 53; Bian et al. 2014: 384.

#### Measurements.

(length in mm)

**Apotrechus parvospinus:** Measurements (length in mm)

	Body	Pronotum	Hind femur	Ovipositor
♂	20.0	3.7	8.6	-
♀	20.0	4.0	9.5	5.0

#### Material.

1♀, Guangxi, Xing’an, Maoer Mountain, 1000m, 22–23.VIII.1992, Xian-Wei Liu & Hai-Sheng Yin leg..

**Figures 22–24.**
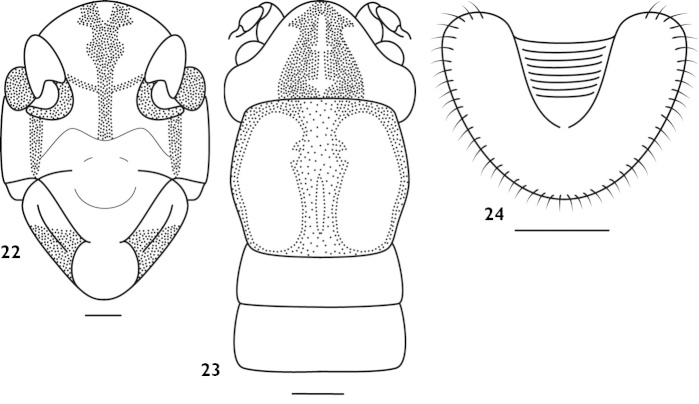
*Apotrechus parvospinus* (Liu & Yin, 2002). **22** head in frontal view **23** head and pronotum in dorsal view **24** subgenital plate of female in ventral view. Scale: 1 mm.

#### Distribution.

China: Guangxi.

### 7. Apotrechus transversus

Taxon classificationAnimaliaOrthopteraGryllacrididae

Liu et al., 2010

urn:lsid:orthoptera.speciesfile.org:TaxonName:73811

Figs 25–29

Apotrechus transversus : Liu et al. 2010: 65, figs 8a–c; Guo and Shi 2012: 53; Bian et al. 2014: 384.

#### Measurements.

(length in mm)

**Apotrechus transversus:** Measurements (length in mm)

	Body	Pronotum	Hind femur	Ovipositor
♂	14.0	3.0	7.0	-
♀	19.0–20.0	3.5–3.8	7.5–8.0	5.0–6.0

#### Material.

1♂1♀, Zhejiang, Longquan, Fengyanshan, 1400m, 27.VII.2007, Qiang Fu leg.; 2♀♀, Zhejiang, Longquan, Fengyanshan, Huangmaojian, 1500–1900m, 31.VII–2.VIII.2008, Xian-Wei Liu & Wen-Xuan Bi.

**Figures 25–29.**
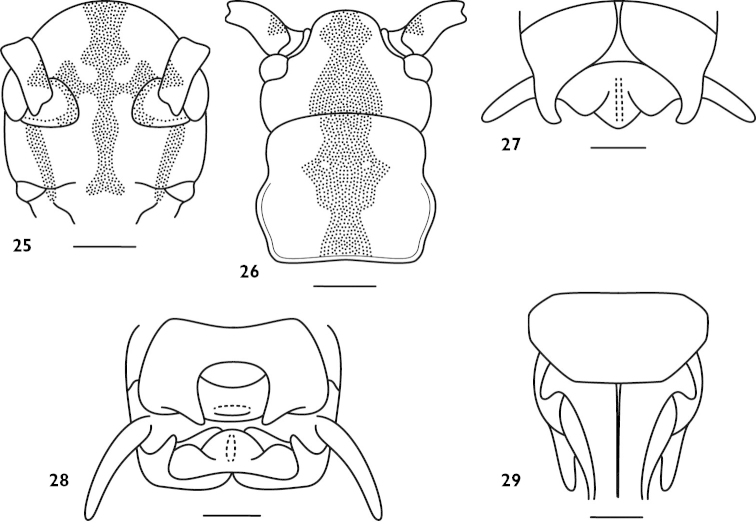
*Apotrechus transversus* Liu et al., 2010. **25** head in frontal view **26** head and pronotum in dorsal view **27** end of male abdomen in dorsal view **28** end of male abdomen in caudal view **29** end of female abdomen in ventral view. Scale: 1 mm.

#### Distribution.

China: Zhejiang.

### 8. Apotrechus digitatus

Taxon classificationAnimaliaOrthopteraGryllacrididae

Liu & Bi, 2008

urn:lsid:orthoptera.speciesfile.org:TaxonName:21788

Figs 30–34

Apotrechus digitatus : Liu and Bi 2008: 12, figs 1–5; Guo and Shi 2012: 53; Bian et al. 2014: 381.

#### Measurements.

(length in mm)

**Apotrechus digitatus:** Measurements (length in mm)

	Body	Pronotum	Hind femur	Ovipositor
♂	15.0	4.0	9.0	-
♀	19.0	4.5	9.0	5.5

#### Material.

1♀1♂, Guizhou, Leigong Mountain, 1620–2178m, 2.VIII.2004, Kai Yan & De-Yan Ge leg.

**Figures 30–34.**
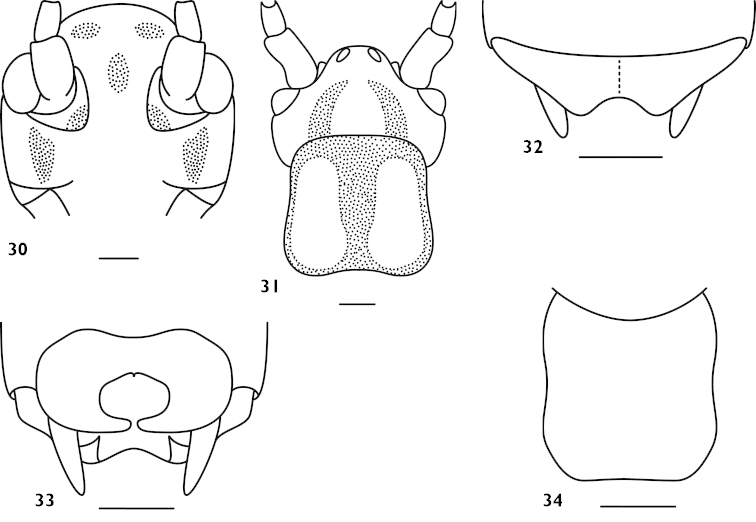
*Apotrechus digitatus* Liu & Bi, 2008. **30** head in frontal view **31** head and pronotum in dorsal view **32** end of male abdomen in dorsal view **33** end of male abdomen in ventral view **34** subgenital plate of female in ventral view. Scale: 1 mm.

#### Distribution.

China: Guizhou.

### 9. Apotrechus bilobus

Taxon classificationAnimaliaOrthopteraGryllacrididae

Guo & Shi, 2012

urn:lsid:orthoptera.speciesfile.org:TaxonName:73812

Figs 35–37

Apotrechus bilobus : Guo and Shi 2012: 55, figs 1–5, 12–13, 17–18; Bian et al. 2014: 380–381.

#### Measurements.

(length in mm)

**Apotrechus bilobus:** Measurements (length in mm)

	Body	Pronotum	Hind femur	Ovipositor
♂	15.0–17.5	3.5	8.0–9.5	-
♀	20.0–22.0	4.0–4.2	9.0	4.7–5.0

#### Material.

1♂, Zhejiang, Lin’an, Xitianmu Mountain, 1140m, 28.VII–2.IX.2010, Hui Pan leg.

**Figures 35–37.**
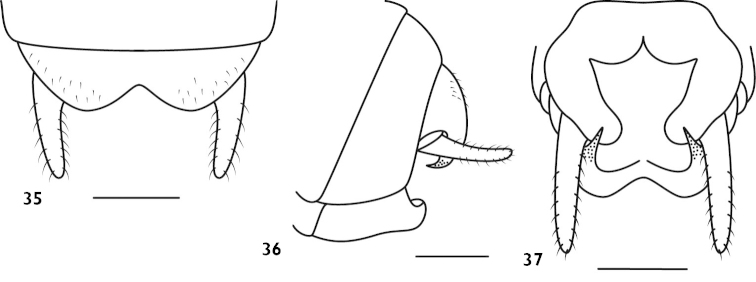
*Apotrechus bilobus* Guo & Shi, 2012. **35** end of male abdomen in dorsal view **36** end of male abdomen in lateral view **37** end of male abdomen in ventral view. Scale: 1 mm.

#### Distribution.

China: Zhejiang.

## Supplementary Material

XML Treatment for Apotrechus

XML Treatment for Apotrechus trilobus

XML Treatment for Apotrechus quadratus

XML Treatment for Apotrechus nigrigeniculatus

XML Treatment for Apotrechus fallax

XML Treatment for Apotrechus truncatolobus

XML Treatment for Apotrechus parvospinus

XML Treatment for Apotrechus transversus

XML Treatment for Apotrechus digitatus

XML Treatment for Apotrechus bilobus
